# Development of an advanced diagnostic concept for intestinal inflammation: molecular visualisation of nitric oxide in macrophages by functional poly(lactic-*co*-glycolic acid) microspheres

**DOI:** 10.3762/bjnano.8.163

**Published:** 2017-08-08

**Authors:** Kathleen Lange, Christian Lautenschläger, Maria Wallert, Stefan Lorkowski, Andreas Stallmach, Alexander Schiller

**Affiliations:** 1 Jena University Hospital, Department of Internal Medicine IV (Gastroenterology, Hepatology, Infectiology), Am Klinikum 1, 07743 Jena, Germany; 2 Atherothrombosis and Vascular Laboratory, Baker Heart and Diabetes Institute, Melbourne, Australia; 3 Competence Cluster for Nutrition and Cardiovascular Health (nutriCARD) Halle–Jena–Leipzig and Friedrich Schiller University of Jena, Institute of Nutrition, Department of Nutritional Biochemistry, Dornburger Straße 25, 07743 Jena, Germany; 4 Friedrich Schiller University Jena, Institute for Inorganic and Analytical Chemistry, Humboldtstr. 8, 07743 Jena, Germany

**Keywords:** functional imaging, intestinal inflammation, microparticle, molecular imaging, nitric oxide

## Abstract

We here describe a new approach to visualise nitric oxide (NO) in living macrophages by fluorescent NO-sensitive microspheres based on poly(lactic-*co*-glycolic acid) (PLGA). PLGA microspheres loaded with NO550 dye were prepared through a modified solvent-evaporation method. Microparticles were characterized by a mean hydrodynamic diameter of 3000 nm, zeta potential of −26.000 ± 0.351 mV and a PDI of 0.828 ± 0.298. Under abiotic conditions, NO release was triggered through UV radiation (254 nm) of 10 mM sodium nitroprusside dehydrate (SNP). After incubation, AZO550 microspheres exhibited an about 8-fold increased emission at 550 nm compared to NO550 particles. For biotic NO release, RAW 264.7 murine macrophages were activated with lipopolysaccharide (LPS) of *Salmonella typhimurium*. After treatment with NO550 microparticles, only activated cells caused a green particle fluorescence and could be detected by laser scanning microscopy. NO release was confirmed indirectly with Griess reaction. Our functional NO550 particles enable a simple and early evaluation of inflammatory and immunological processes. Furthermore, our results on particle-based NO sensing and previous studies in targeting intestinal inflammation via (PLGA)-based microspheres demonstrate that an advanced concept for visualizing intestinal inflammation is tangible.

## Introduction

Inflammation and malignancies are fundamental aspects of many human diseases. Nitric oxide (NO) has been proposed to be an important mediator of inflammation and carcinogenesis. Chronic inflammation, as found in inflammatory bowel diseases, seems to be maintained by high levels of nitric oxide (NO) due to an abnormal immune response against endogenous flora and luminal antigens in genetically susceptible individuals. High levels of NO become noxious to mucosal tissue. As NO levels even correlate with severity of disease, imaging of mucosal NO concentrations improves the assessment of disease activity and even may contribute to predict disease progression before mucosal damage continues. The visualisation of molecular processes that drive mucosal inflammation is of great interest in life sciences. NO is synthesised and released on demand, it is not stored and is highly diffusible. In vivo detection of NO in real time is difficult, because NO rapidly diffuses and reacts with cellular components. NO quantification with chemiluminescence or amperometry is often complicated by low spatio-temporal resolution and complex experimental set-ups prone to interferences [1]. Hence, the development of highly sensitive fluorescent sensors for NO imaging may improve its visualisation in vivo significantly [2]. Furthermore, molecular imaging of NO enables a better visualisation of intestinal functionalities, including irregular mucosal patterns and vascular lesions [3].

We developed a novel polymeric microparticle made of biodegradable poly(lactic-*co*-glycolic acid) (PLGA), which accumulates selectively in inflamed mucosa of patients with inflammatory bowel disease without interfering with the healthy mucosa. This approach is based on the epithelial barrier dysfunction of the intestine during intestinal inflammation. The intestinal barrier shows an increased permeability by disabled tight junction proteins, alterations in the thickness and composition of the mucus. Thus, particles penetrate and accumulate only into the inflamed mucosa [4]. Previously, we have shown that polymeric particles penetrate and accumulate selectively within the inflamed mucosa proving that a particle-based approach is feasible [5–6]. Now, we introduce a cutting-edge strategy to visualise NO in living macrophages as first step, and to visualise these cells in NO-mediated intestinal inflammation in vivo by fluorescent particle-based diagnostics in a second step.

Here, we used a NO-sensitive dye, namely NO550, as a model molecule to proof the concept of a particle-based diagnostic as part of an advanced diagnostic concept for detecting intestinal inflammation. NO550 is a chemical sensor for the cellular imaging of NO, while being inert to other reactive oxygen and nitrogen species (ROS/RNS), and is characterized by high specificity and low background signals [7]. We prepared and characterized NO550-loaded PLGA microspheres to study NO in abiotic and biotic experiments. To our knowledge, this is the first time of visualisation of NO at different concentrations via fluorescence-emitting NO550-PLGA microspheres.

## Results and Discussion

### Physicochemical characterisation of microspheres

**Figure 1:**
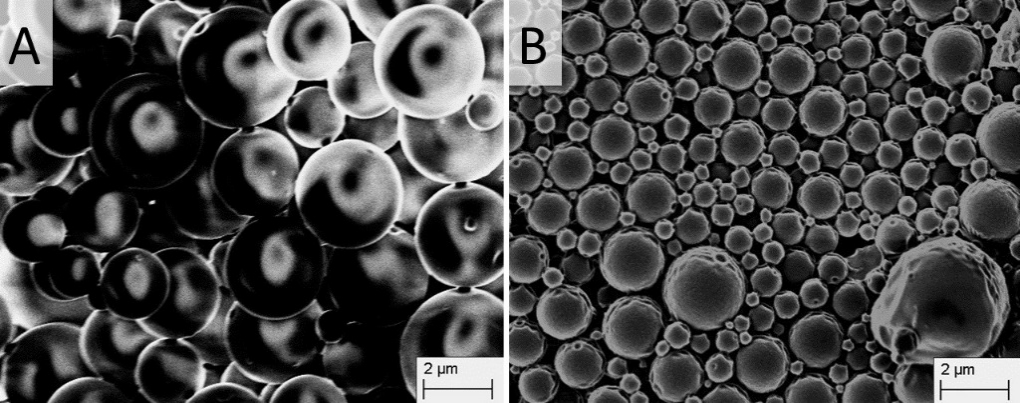
Scanning electron microscopic images of blank (A) and NO550-loaded (B) polymeric microspheres.

The preparation of NO550-loaded microspheres was a reliable and reproducible process (for more experimental data please see Section 1 and Section 2 of Supporting Information File 1). The particles showed a mean hydrodynamic diameter of 3000 nm, a zeta potential of −26.000 ± 0.351 mV and a PDI of 0.828 ± 0.298. Furthermore, NO550-loaded microspheres were characterised by a slightly more irregular surface with small pores compared to blank microspheres (Figure 1). In contrast, blank microspheres showed a mean hydrodynamic diameter of 3000 nm, a zeta potential of −1.250 ± 0.132 mV and a PDI of 0.253 ± 0.042. The blank microspheres are similarly sized spherical particles with smooth, uniform and pore-free surfaces (Figure 1). The influence of NO550 leads to a higher PDI, a shift of the zeta potential and an irregular surface compared to blank microspheres.

### Abiotic nitric oxide sensing studies

**Figure 2:**
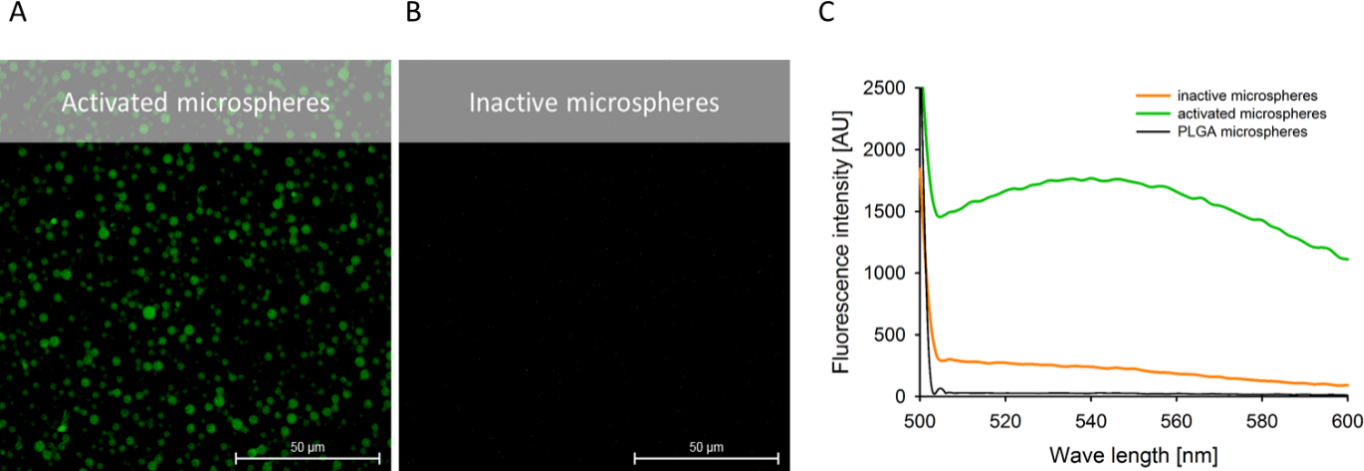
NO-releasing sodium nitroprusside (SNP) leads to light emission of NO550-loaded microspheres. Confocal laser scanning microscopy (CLSM) images of activated (A) and inactivated (B) NO550-loaded polymeric microspheres in DPBS including fluorescence emission spectra (C) of the microspheres are shown. UV-irradiated SNP (10 mM, 2 min, 254 nm) was used for 2 minutes to activate NO550-loaded microspheres. During the incubation with SNP, NO550 is converted to AZO550 that emits green fluorescence at 550 nm.

In this study, we used sodium nitroprusside (SNP) as an exogenous NO donor. SNP in aqueous medium is highly photosensitive and releases NO in a constant manner when irradiated with UV light. This experimental approach allows for abiotic NO sensing studies. After 2 minutes of incubation of NO550-loaded microspheres with UV-irradiated SNP (10 mM, 2 min, 254 nm) we observed an up to 8-fold increased fluorescence signal at 550 nm compared to inactive NO550-loaded microspheres (see Section 3 of Supporting Information File 1 for further experimental data). Thus, NO550-loaded particles could sense abiotic NO by reacting with the encapsulated NO550. The conversion into the fluorescent AZO550 was detected photometrically (Figure 2).

### Biotic nitric oxide sensing studies

**Figure 3:**
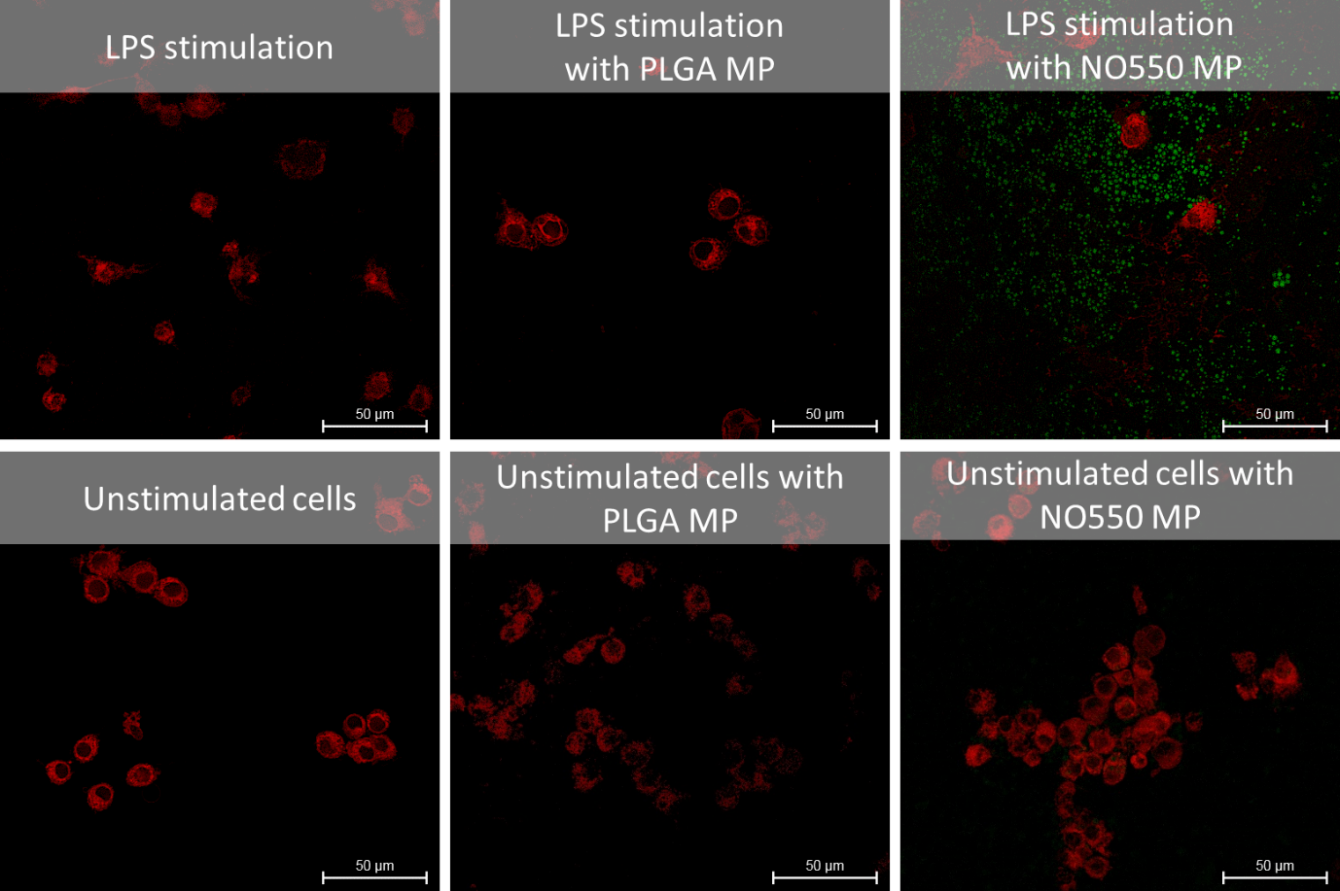
NO550-loaded microspheres detect the inflammatory response of murine macrophage-like RAW 264.7 cells. CLSM images of LPS-stimulated and non-stimulated RAW 264.7 cells are shown. Cells were stained with Cell Mask deep red. The treatment of RAW 264.7 cells with LPS increased formation and release of NO which in turn converted NO550 into AZO550 within the NO550-loaded microspheres. NO formation is visualised as green fluorescence signal during LPS stimulation.

**Figure 4:**
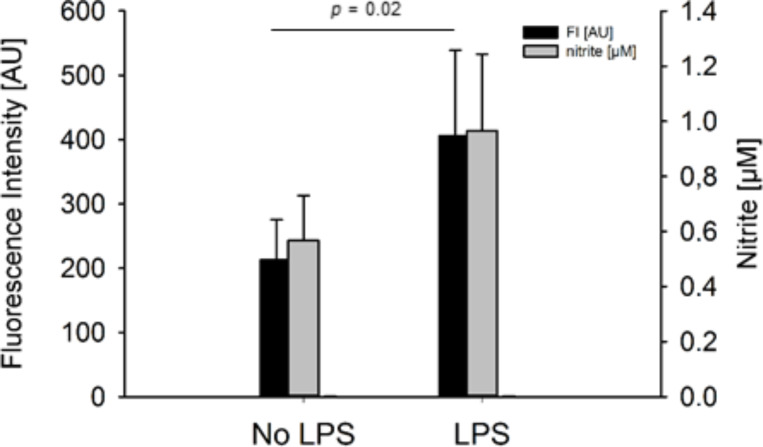
Quantification of NO release with NO550-loaded microspheres in inflamed cells. LPS-stimulated murine RAW 264.7 macrophages led to significantly higher fluorescence intensity (FI) signals using NO550-loaded microspheres than in unstimulated cells (*p* = 0.02). The signal coincide with nitrite concentrations estimated by Griess reaction. AU: arbitrary units.

In our studies, we used the murine RAW264.7 macrophage cell line as an established model to analyse the endogenous formation of NO. This experimental approach allows for biotic NO sensing studies with NO550-loaded microspheres. LPS from *Salmonella typhimurium* was used to induce NO production in these macrophages. The pathogen *Salmonella typhimurium* causes severe intestinal inflammations, in some cases even leading to bacteraemia. LPS increased cellular production and release of NO in RAW264.7 macrophages up to 50 µM after 24 h of stimulation compared to non-stimulated macrophages. The incubation (1 h) of LPS-treated RAW264.7 with microspheres resulted in a slight increase in NO formation in the range of about 1 µM, while native untreated cells displayed basal NO levels of approximately 0.6 µM. Microscopic investigation of untreated macrophages confirmed this by a low fluorescence signal of NO550-loaded microspheres (Figure 3). In contrast, NO550-loaded microspheres revealed in LPS-stimulated macrophages a strong fluorescence signal at 550 nm. Due to the polymeric encapsulation of NO550, no fluorescent background was observed. The microspheres showed a clear round shape with homogenous fluorescence. Some smaller microspheres were located within macrophages, likely because of endocytic uptake by the RAW264.7 macrophages (Figure 3). These results clearly show that NO550-loaded particles can be used to sense biotic NO by the reaction of NO with the encapsulated NO550. The conversion of NO550 into the fluorescent AZO550 molecule can be detected chemically and spectrometrically. Furthermore, the fluorescence intensity of the microspheres in the supernatants of the cells can be measured using a plate reader. In the same supernatants, we determined NO release using the Griess reaction (see Section 4 and Section 5 of Supporting Information File 1 for experimental data). These experiments showed that the fluorescence intensity of the supernatants coincided with the amount of nitrite in the supernatant of LPS-stimulated cells as determined by the Griess reaction (Figure 4).

## Conclusion

We demonstrated the possibility of molecular imaging of NO at different concentrations and under different conditions using NO550-loaded PLGA microspheres in living macrophages. NO550 is converted by NO into an azo dye, which emits green fluorescence in an NO concentration-dependent manner. Thus, this approach provides a novel approach for the early spatio-temporal evaluation of inflammatory processes in IBD. The intestinal distribution and signal intensity of the microspheres can be easily analysed by fluorescence-based microscopy. A next step will be to adopt our approach for the use with fluorescence-based endoscopy and for the visualisation of NO release in mucosal biopsies obtained from patients with chronic intestinal inflammation. Furthermore, our results on particle-based NO-sensing and previous works in targeting intestinal inflammation via PLGA microspheres demonstrate that an advanced concept for visualizing intestinal inflammation is tangible.

## Supporting Information

File 1 Additional experimental data.

## References

[1] Hetrick E M, Schoenfisch M H (2009). Annu Rev Anal Chem.

[2] Li H, Wan A (2015). Analyst.

[3] Schmidt C, Lautenschläger C, Petzold B, Sakr Y, Marx G, Stallmach A (2013). Br J Anaesth.

[4] Lautenschläger C, Schmidt C, Fischer D, Stallmach A (2014). Adv Drug Delivery Rev.

[5] Lautenschläger C, Schmidt C, Lehr C-M, Fischer D, Stallmach A (2013). Eur J Pharm Biopharm.

[6] Schmidt C, Lautenschlaeger C, Collnot E-M, Schumann M, Bojarski C, Schulzke J-D, Lehr C-M, Stallmach A (2013). J Controlled Release.

[7] Ghebremariam Y T, Huang N F, Kambhampati S, Volz K S, Joshi G G, Anslyn E V, Cooke J P (2014). J Vasc Res.

