# Supplementary material for: Development of an advanced diagnostic concept for intestinal inflammation: molecular visualisation of nitric oxide in macrophages by functional poly(lactic-co-glycolic acid) microspheres
Source: Beilstein J Nanotechnol. 2017 Aug 8;8:1637–41. doi: 10.3762/bjnano.8.163 (PMC5564270; doi:10.3762/bjnano.8.163)
Supplement: File 1 — Additional experimental data. [file Beilstein_J_Nanotechnol-08-1637-s001.pdf]

Supporting Information

for

Development of an advanced diagnostic concept for intestinal inflammation: molecular visualisation of nitric oxide in macrophages by functional poly(lactic-*co*-glycolic acid) microspheres

Kathleen Lange[*,‡,§,1], Christian Lautenschläger[‡,1], Maria Wallert[2], Stefan Lorkowski[3], Andreas Stallmach[1] and Alexander Schiller[4]

Address: [1]Jena University Hospital, Department of Internal Medicine IV (Gastroenterology, Hepatology, Infectiology), Am Klinikum 1, 07743 Jena, Germany; [2]Atherothrombosis and Vascular Laboratory, Baker Heart and Diabetes Institute, Melbourne, Australia; [3]Competence Cluster for Nutrition and Cardiovascular Health (nutriCARD) Halle–Jena–Leipzig and Friedrich Schiller University of Jena, Institute of Nutrition, Department of Nutritional Biochemistry, Dornburger Straße 25, 07743 Jena, Germany and [4]Friedrich Schiller University Jena, Institute for Inorganic and Analytical Chemistry, Humboldtstr. 8, 07743 Jena, Germany

Email: Kathleen Lange* - kathleen.lange@med.uni-jena.de

* Corresponding author
‡ Equal contributors
§ Phone: +49 3641 9324641

Additional experimental data

1 Preparation of NO550-loaded microspheres

The preparation of NO550-loaded PLGA particles was based on a modified solvent evaporation method [1]. PLGA (RESOMER RG 502, M_W = 7000–17000, lactide:glycolide 50:50) was dissolved in ethyl acetate as organic phase. The aqueous phase was prepared by dissolving 1% polyvinyl alcohol (PVA) in MilliQ water. NO550 (kindly gifted by Professor Eric V. Anslyn, University of Texas) was dissolved in dimethyl sulfoxide (DMSO) and was added to the organic (0.5 mg/mL) and aqueous phase (0.5 mg/mL). The organic phase was added dropwise to aqueous phase under stirring at 400 rpm. The resultant organic/water emulsion was homogenized for the preparation of microspheres (5000 rpm, 15 min).

2 Characterisation of NO550-loaded microspheres

The mean hydrodynamic diameters and the zeta potential (ZP) of the particles were determined using a Zetasizer Nano ZS instrument. All experiments were performed in at least three independent replicates (five runs per sample), and mean values were calculated. ZP measurements were performed in deionized water by determining the electrophoretic mobility at 25 °C in DTS1060 capillary cells. The hydrodynamic diameter and the polydispersity index (PDI) were measured via photon correlation spectroscopy (PCS) in deionized water using low-volume cuvettes (ZEN 0112), a temperature of 25 °C and a scattering angle of 173°. Data were analyzed using Zetasizer v7.11 software and the refractive index (1.33) and viscosity (0.88 mPa·s) of distilled water at 25 °C. Scanning electron microscopy (SEM) imaging of microspheres was performed using Carl Zeiss ULTRA 55.

3 Abiotic NO sensing studies

NO was generated using a 10 mM sodium nitroprusside dihydrate (SNP) solution in 0.9% NaCl. NO release was triggered through UV light (254 nm, 2 min). Then, NO550-loaded microspheres were added and incubated for 5 min. The fluorescence intensity of all incubated microspheres was measured in a plate reader. For single particle analysis, microspheres were detected by confocal laser scanning microscopy (CLSM).

4 Biotic NO sensing studies

Murine RAW264.7 macrophages were cultivated in DMEM containing 4.5 g/L glucose, L-glutamine and pyridoxine and supplemented with 10% fetal calf serum (FCS) and 1% penicillin/streptomycin/glutamine. Cells were cultivated at 37 °C, 95% humidity and under 5% CO_2 atmosphere and maintained at 50–60% sub-confluence. For NO sensing, cells were seeded in 24-Well plates for quantitative NO and nitritre detection and on 8-Well glass chamber slides for CLSM microsocopy. Cells were stimulated with 100 ng/mL lipopolysaccharide (LPS) obtained from *Salmonella typhimurium* for 24 h. Then, the culture supernatant was removed, cells were rinsed once and microspheres were added (0.4 mg/cm^2) in 50 µL of Dulbecco´s phosphate buffered saline (DPBS). After 1 h incubation, the supernatant was centrifuged and collected. The total fluorescence intensity of supernatant was measured in a plate reader. The quantification of nitrite as a result from released NO was performed by Griess reaction. Therefore, culture supernatant was centrifuged (5 min and 900 rpm) and 1% sulfonilamid and 0.1% *N*-(1-naphtyl)ethylenediamine were sequentially added at a final ratio of 1:3 and incubated for 5 min (first described by Griess 1879). Light adsorption at 533 nm was measured photometrically. Nitrite concentrations were calculated using a standard curve derived from serial diluted 0.1 mM sodium nitrite. For fluorescence analyses of single microspheres and macrophages by CLSM, the murine macrophages were stained with CellMask Deep Red.

5 Statistical analysis

Statistical analysis was performed using Sigma Plot Version 12.5 Values are given as mean ± SD. One way ANOVA with Tukey's post hoc correction was used for multiple comparisons, a t-test was used if only two groups were compared. Significance was considered at $p < 0.05$.

References

1. Lautenschläger, C.; Schmidt, C.; Lehr, C.-M.; Fischer, D.; Stallmach, A. *Eur. J. Pharm. Biopharm.* **2013,** *85,* 578–586. doi:10.1016/j.ejpb.2013.09.016
